# Integrative analysis of single-cell and bulk RNA-sequencing data revealed T cell marker genes based molecular sub-types and a prognostic signature in lung adenocarcinoma

**DOI:** 10.1038/s41598-023-50787-w

**Published:** 2024-01-10

**Authors:** Yueling Peng, Yafang Dong, Qihui Sun, Yue Zhang, Xiangyang Zhou, Xiaoyang Li, Yuehong Ma, Xingwei Liu, Rongshan Li, Fengjie Guo, Lili Guo

**Affiliations:** 1 Shanxi Provincial Key Laboratory of Kidney Disease, Shanxi Provincial People’s Hospital of Shanxi Medical University, Taiyuan, 030012 Shanxi China; 2 Department of Nephrology, Shanxi Provincial People’s Hospital (Fifth Hospital of Shanxi Medical University), Taiyuan, 030012 China; 3 Department of Pathology and Pathophysiology, School of Basic Medical Sciences, Shanxi Medical University, Taiyuan, 030001 China; 4 Department of Cell Biology, School of Basic Medicine, Shanxi Medical University, Taiyuan, 030001 China; 5 South China University of Technology, Guangzhou, 510006 China; 6 Tianjin Key Laboratory of Lung Cancer Metastasis and Tumor Microenvironment, Tianjin Lung Cancer Institute, Tianjin Medical University General Hospital, Tianjin, 300052 China

**Keywords:** Biomarkers, Cancer

## Abstract

Immunotherapy has emerged as a promising modality for addressing advanced or conventionally drug-resistant malignancies. When it comes to lung adenocarcinoma (LUAD), T cells have demonstrated significant influence on both antitumor activity and the tumor microenvironment. However, their specific contributions remain largely unexplored. This investigation aimed to delineate molecular subtypes and prognostic indicators founded on T cell marker genes, thereby shedding light on the significance of T cells in LUAD prognosis and precision treatment. The cellular phenotypes were identified by scrutinizing the single-cell data obtained from the GEO repository. Subsequently, T cell marker genes derived from single-cell sequencing analyses were integrated with differentially expressed genes from the TCGA repository to pinpoint T cell-associated genes. Utilizing Cox analysis, molecular subtypes and prognostic signatures were established and subsequently verified using the GEO dataset. The ensuing molecular and immunological distinctions, along with therapy sensitivity between the two sub-cohorts, were examined via the ESTIMATE, CIBERSORT, and ssGSEA methodologies. Compartmentalization, somatic mutation, nomogram development, chemotherapy sensitivity prediction, and potential drug prediction analyses were also conducted according to the risk signature. Additionally, real-time qPCR and the HPA database corroborated the mRNA and protein expression patterns of signature genes in LUAD tissues. In summary, this research yielded an innovative T cell marker gene-based signature with remarkable potential to prognosis and anticipate immunotherapeutic outcomes in LUAD patients.

## Introduction

For several decades, lung cancer has remained the most commonly diagnosed cancer worldwide, responsible for more than 20% of all cancer deaths globally^1^. Pulmonary adenocarcinoma (LUAD) constitutes the predominant histological subtype, encompassing close to 45% of total lung carcinoma incidences^2,3^. Notwithstanding advancements in, and the utilization of, a confluence of therapeutic approaches and personalized treatments for LUAD, the 5-year overall survival rate associated with this malignancy continues to be under 25%, which is an unsatisfactory result^4^. The recent adoption of immunotherapies aimed at immune checkpoints has significantly advanced clinical benefits and has subsequently altered the treatment landscape for LUAD^5,6^. Immune checkpoint inhibitors (ICIs) are emerging as a promising strategy for the treatment of LUAD, due to their ability to enhance the body's natural ability to fight tumors, compared to traditional treatment modalities^7,8^. Unfortunately, only a small number of LUAD patients can benefit from immune checkpoint inhibitor (ICI) therapy^9^. Therefore, it is of pressing importance to discover fitting biomarkers and establish relevant prediction models to effectively estimate prognosis and therapeutic outcomes in LUAD.

The tumor microenvironment (TME) is a complex biological system encompassing tumor cells and their surrounding elements, such as immune cells, mesenchymal cells, endothelial cells, the extracellular matrix, and various intercellular communication molecules like cytokines, chemokines, and growth factors^10–12^. These components interact with one another, giving rise to the highly intricate and dynamic nature of the lung adenocarcinoma tumor microenvironment, which collectively contributes to tumor growth and progression^13,14^. The TME profoundly influences T cell activity, function, and effects, which play a pivotal role in antitumor immune responses^15–17^. Although adaptive T cell responses have been extensively studied in antitumor immunity, the role of innate immune cells remains underexplored. The existence and activation state of T cells hold potential as prognostic indicators in NSCLC^18^. However, the CD8+ T cell differentiation trajectory in NSCLC could hamper the sensitivity of CD8+ T cells to immune checkpoint therapy, potentially leading to ICB failure in T cell-infiltrated NSCLC^19^. Understanding the underlying mechanisms of T cell immune factors is crucial for overcoming drug resistance in LUAD therapy^20^. Considering the scarcity of research on the antitumor immune effects of LUAD pertaining to T cells, examining the gene expression patterns and their association with prognosis and therapeutic outcomes is of paramount importance.

Single-cell sequencing is a high-throughput technique that analyzes an individual cell's genome, transcriptome, or epigenome^21,22^. This powerful tool enables researchers to investigate cellular heterogeneity and cell-to-cell interactions^23,24^. With its crucial role in identifying new therapeutic targets, examining cellular heterogeneity, and monitoring treatment efficacy and resistance in targeted tumor therapy and immunotherapy, an increasing number of studies are personalizing treatment by analyzing tumor cell and immune cell interactions to predict patient response to immunotherapy^25–27^. Our study involved a comprehensive assessment of scRNA-seq and bulk RNA-seq data obtained from LUAD samples, aiming to discern T cell marker genes and establish prognostic signatures (Supplementary Figure 1). We further validated the signature's predictive utility using the GEO cohort. Additionally, we examined variations in immune checkpoints expression levels, tumor mutational burden (TMB), and chemotherapy response. These findings have the potential to yield therapeutic targets and predictive indicators for LUAD.

## Results

### Identifying T-cell marker genes expression profiles

**Figure 1:**
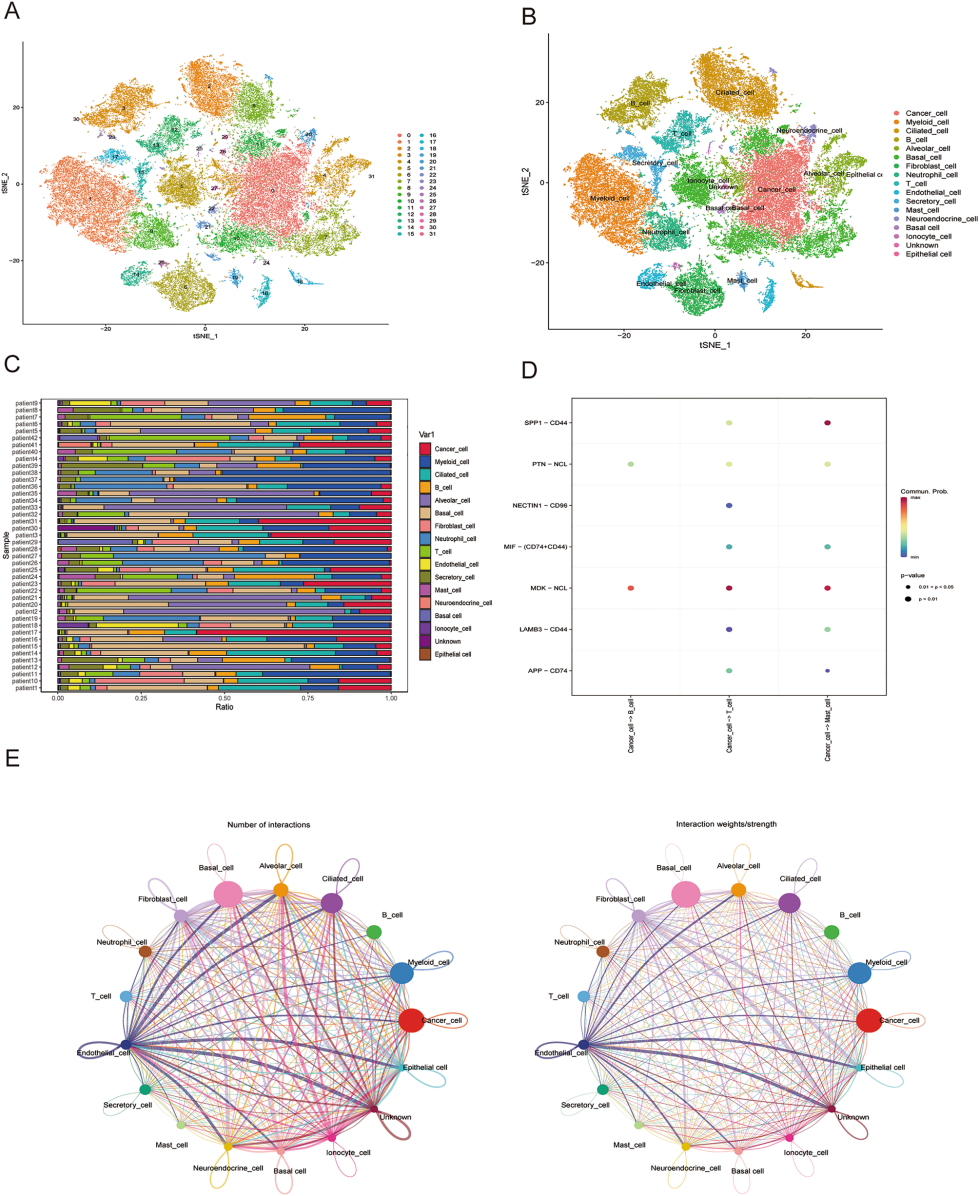
Identification of the LUAD-associated cell subtypes. (**A**) t-SNE plot classifying cell clusters based on scRNA sequencing data. (**B**) t-SNE plot identifying the various cell subtypes. (**C**) Proportions of different cell types. (**D**) Ligand‒receptor pairs for all signalling pathways between cancer cells and immune cells. (**E**) Intercellular communication network of 17 cell subtypes.

Drawing on single cell profiles from GSE148071, we extracted gene expression matrix encompassing 60,288 cells derived from 42 original LUAD samples, subsequently subjecting them to further scrutiny (Fig. 1A). The harmony method facilitated dimensionality reduction, revealing 17 distinct cell clusters—including Cancer cell, Myeloid cell, B cell, Ciliated cell, Alveolar cell, Basal cell, Fibroblast cell, Neutrophil, Endothelial cell, T cell, Secretory cell, Mast cell, Neuroendocrine cell, Basal cell, Ionocyte cell, and Epithelial cell—each identified by their characteristic marker genes (Fig. 1B, Supplementary Table 1). Additionally, we observed marked discrepancies in T cell distribution across various LUAD patient specimens, leading us to identify LUAD-associated T cell marker genes (Fig. 1C). Application of the "CellChat" approach unveiled a substantial degree of connectivity between different cell types (Fig. 1E). Ultimately, we constructed a signaling pathway map incorporating three Immunocytes (Mast, B, and T cell) and cancer cells (Fig. 1D).

### Identification of differential T cell marker genes and biological function enrichment analysis

**Figure 2:**
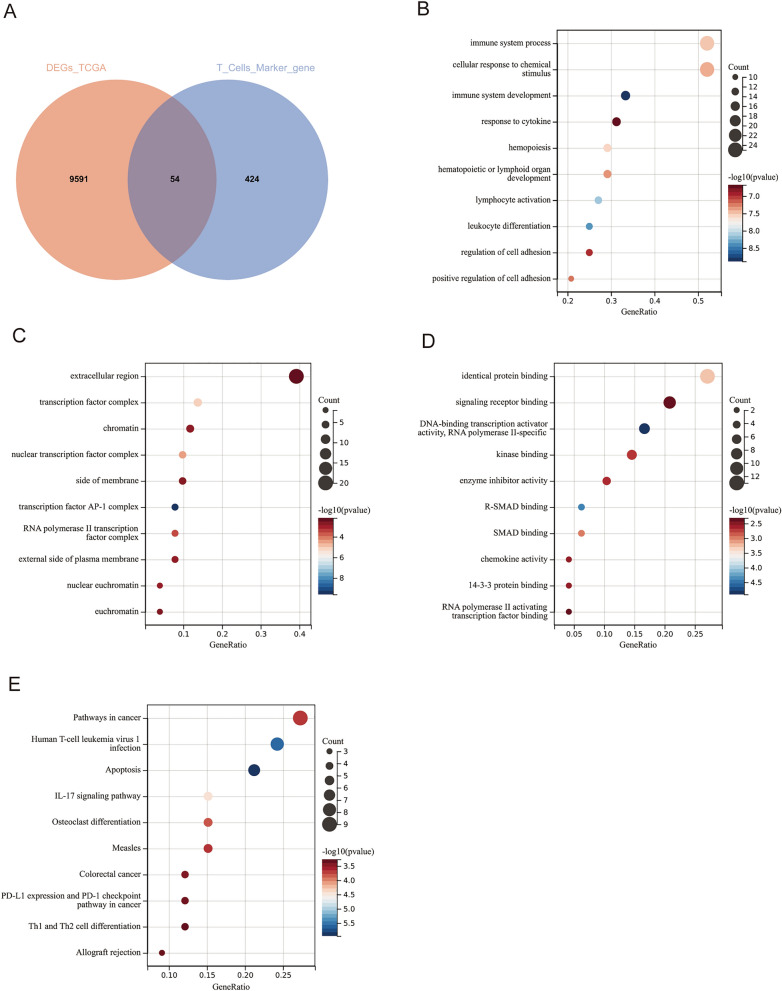
Enrichment analysis (**A**) Venn diagram showing the overlap of genes between T cell marker genes and the DEGs in TCGA data. (**A**) BP, biological process; (**B**) CC, cellular component; (**C**) MF, molecular function. (**D**) KEGG pathway enrichment analysis of the differential T cell marker genes.

Through the examination of single-cell samples, we procured 578 T cell marker genes. Following the results of the comparison of tumor and healthy lung tissues from the TCGA database, we identified 9645 DEGs. The intersection of these genes was deemed to be differential T cell marker genes and utilized for subsequent downstream analyses (Fig. 2A). According to GO analysis, the biological processes were mainly enriched in immune system process, cellular response to chemical stimulus, and immune system development (Fig. 2B). Cellular components were primarily concentrated in extracellular region, transcription factor complex, and chromatin (Fig. 2C). In the molecular function category, differential T cell marker genes were predominantly associated with identical protein binding, signaling receptor binding, and DNA-binding transcription activator activity, RNA polymerase II-specific (Fig. 2D). In the context of KEGG analysis, the results demonstrated that these genes were significantly associated with the pathways in cancer, human T-cell leukemia virus 1 infection, apoptosis pathway and IL-17 signaling pathway (Fig. 2E). In summary, the aforementioned findings indicate that immune-related functions are closely connected to genes within the overlapping set.

### Identification of molucelar subtypes and a correlation analysis of subtypes with tumour immunological milieu and tumourigenic grades

**Figure 3:**
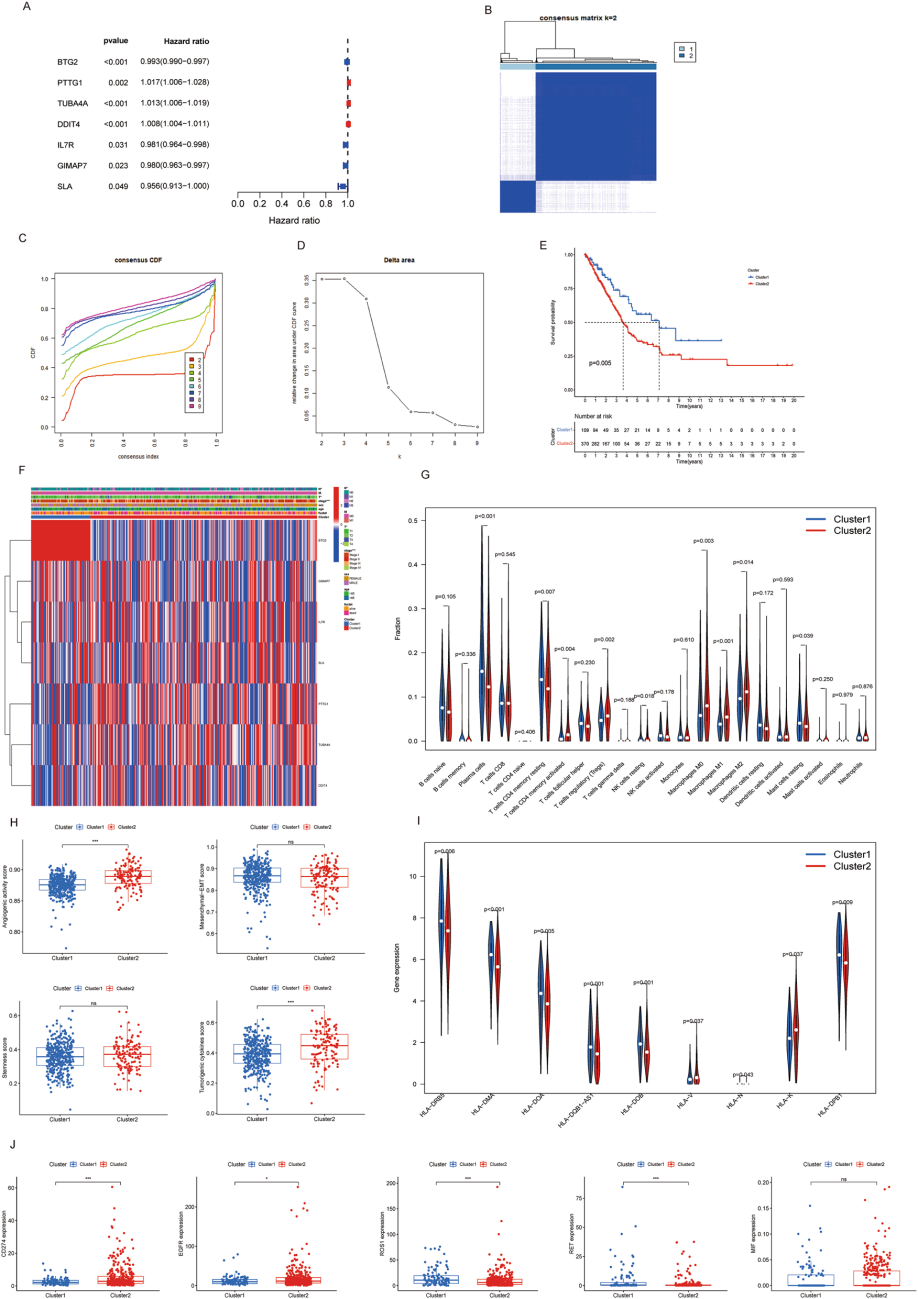
(**A**) Forest plot of seven prognostic-related deferentially expressed T cell marker genes through univariate Cox analysis. (**B**) Consensus clustering matrix when k = 2. (**C**) Consensus clustering CDF with k valued 2 to 9. (**D**) Relative change in area under CDF curve for k = 2. (**E**) KM curve of the survival difference between cluster 1 and cluster 2. (**F**) Heatmap of the seven genes between the two subtypes and the correlations of the clusters and clinical parameters. (**G**) Immune cell infiltration patterns based on CIBERSORT. (**H**) Angiogenic activity, mesenchymal-EMT, tumourigenic cytokines and stemness scores. (**I**) The expression of MHC molecules. (**J**) Five common immunoinhibitors expression levels between the two clusters.

Through univariate Cox analysis, we identified seven prognosis-associated genes, with PTTG1, TUBA4A, and DDIT4 serving as protective factors and BTG2, IL7R, GIMAP7, and SLA as risk factors (Fig. 3A). We employed these seven prognosis-related genes for molucelar subtype analysis, resulting in the optimal clustering of LUAD patients into two subgroups, characterized by promising internal coherence and constancy (Fig. 3B–D). Moreover, Cluster 1 showed a favorable prognosis than Cluster 2 according to our data (Fig. 3E). The heatmap revealed discrepancies in the two clusters' gene expression and their strong connection with clinicopathological factors such stage, N stage, and T stage, though no significant differences were observed in sex, age, and M stage (Fig. 3F). The CIBERSORT algorithm indicated significant differences in infiltrating immunocytes; T cells CD4 memory resting were substantially decreased in Cluster 2, whereas T cells CD4 memory activated and T cells regulatory (Tregs) were significantly increased in Cluster 2 (Fig. 3G). Moreover, angiogenic activity and tumorigenic cytokines were markedly higher in Cluster 2 (Fig. 3H). We also observed that Cluster 1 was associated with elevated expression of numerous MHC molecules (Fig. 3I). As the immune cells infiltration situation differed significantly between the sub-types, we assessed the association with the major immunological checkpoints in LUAD treatments. Cluster 2 exhibited enhanced expression of EGFR and PD-L1, and reduced expression of ROS1 and RET (Fig. 3J). This comprehensive approach allowed us to better understand the complex interplay among these factors, thereby providing valuable insights into tumor progression and potential therapeutic targets.

### Construction and validation of an T cell markers signature

**Figure 4:**
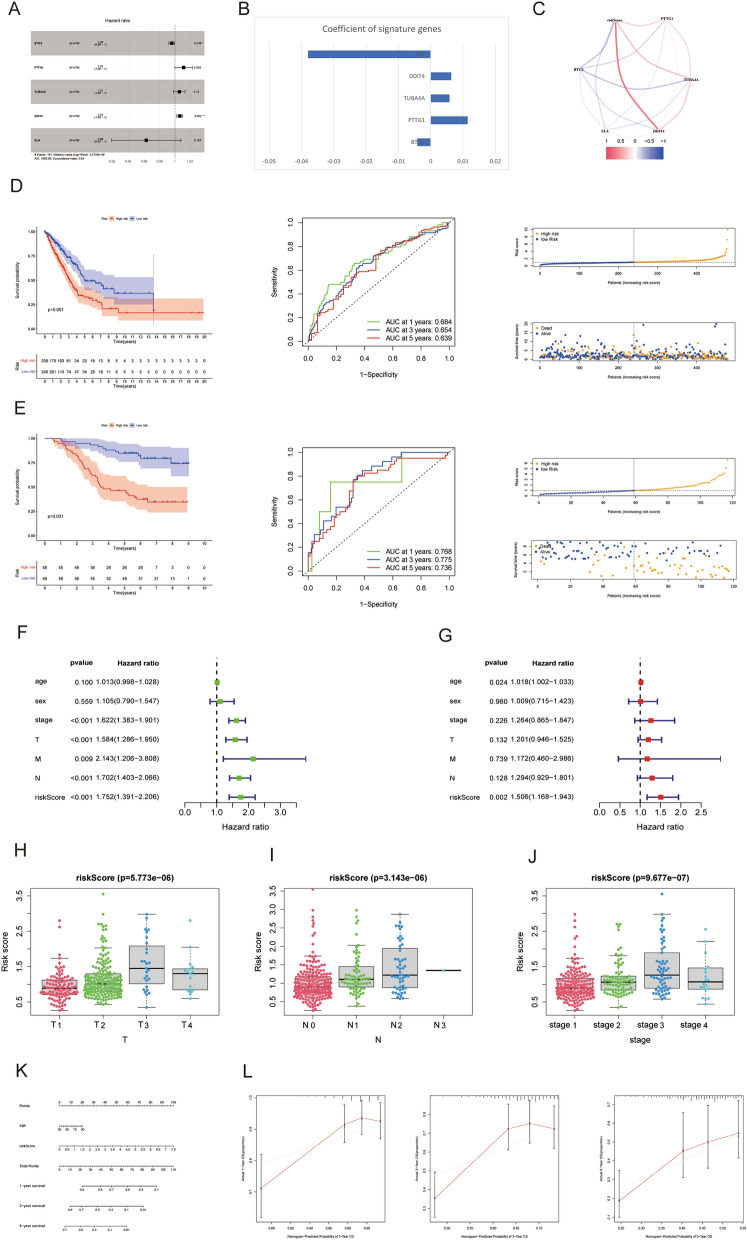
(**A**) Forest plot of the five genes selected in the signature through multivariate Cox analysis. (**B**) Coefficients of the five genes included in the signature. (**C**) The correlations between the signature and the five genes. (**D**) Survival analysis, survival status accompanied with the risk score and ROC analysis in TCGA data and (**E**) GSE13123 data. (**F**,**G**) Univariate and multivariate Cox analysis identified the signature was an independent risk factor for LUAD patients in TCGA. (**H**–**J**) The differences of the risk score between different groups according to clinicopathological features. (**K**) Nomogram based on risk score and age. (**L**) Calibration plots of the nomogram for predicting the probability of 1-, 3- and 5-year survival.

To enhance the specificity of our candidate genes, we performed a multivariate Cox regression analysis and selected five genes for the model (Fig. 4A). The coefficients for individual gene of our signature are displayed (Fig. 4B). The relationship between the calculated risk score and SLA, DDIT4, TUBA4A, PTTG1, and BTG2 is illustrated in Fig. 4C. Patients possessing higher scores exhibited a less favorable prognosis compared to those with lower risk scores, with signature's AUC score measuring 0.684 at 1 year, 0.654 at 3 years, and 0.639 at 5 years (Fig. 4D). We then employed the GSE13213 dataset to verify the reliability and universal applicability of our signature, which demonstrated a promising ability in survival analysis and ROC (Fig. 4E). Furthermore, Fig. 4F,G showed that our signature was also identified as an independent risk factor. We also examined the discrepancy in subgroups between risk scores according to various clinicopathological information. Our findings revealed that patients with T3-4, N2-3, and stage III-IV classifications presented enhanced risk scores, suggesting that higher risk scores were associated with more advanced tumors (Fig. 4H–J). as shown in Fig. 4K, we combined patient age and risk ratings to create a nomogram for estimating 1-, 3-, and 5-year survival prospects in LUAD using the results of multivariate Cox regression analysis. The calibration charts demonstrated a high concordance between the real and anticipated survival times at 1-, 3-, and 5-year intervals (Fig. 4L).

### The evaluation of tumor immunocellular infiltration and immunological checkpoint inhibitors

**Figure 5:**
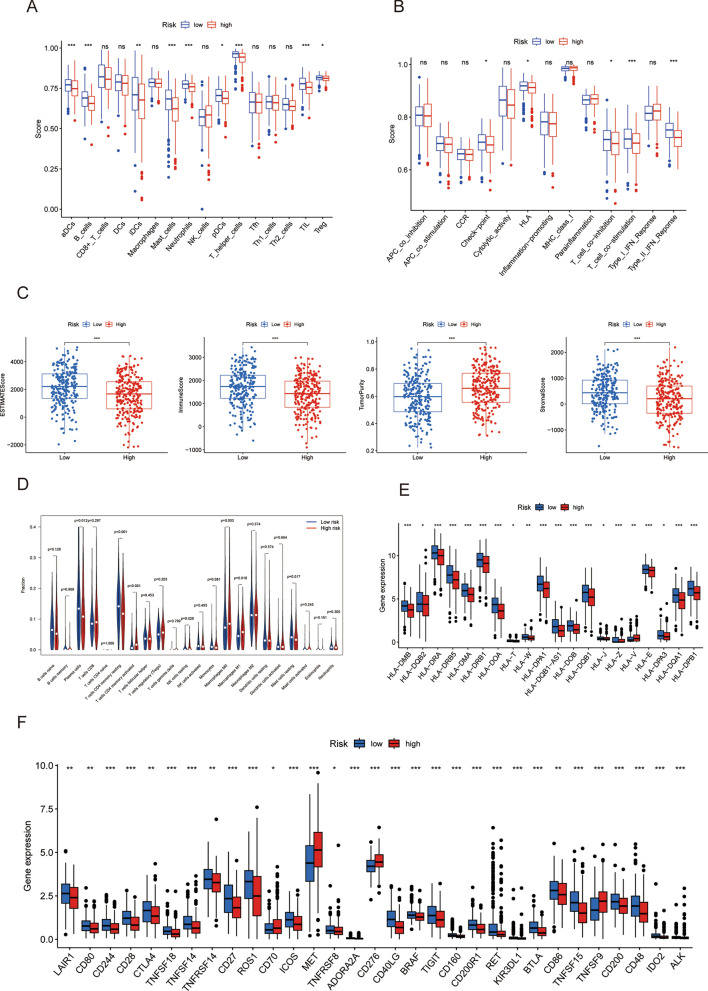
(**A**,**B**) Immune cell infiltration and immune-related functions. (**C**) Immune and stromal scores. (**D**) immune cell infiltration based on CIBERSORT. (**E**) MHC molecules expression patterns. (**F**) Common immunoinhibitors expression levels.

Previous studies have already underscored the crucial importance of the microenvironment in cancer development^28,29^. Accordingly, we conducted a thorough examination of the association between our signature and the tumor immunological microenvironment. Through the utilization of the ssGSEA algorithm, we revealed that the high-risk cohort exhibited decreased infiltration in immune cell and an reduced presence of immune-related pathways in contrast to the low-risk cohort (Fig. 5A,B). Furthermore, the ESTIMATE algorithm showed that high-risk cohort presented reduced ESTIMATE score, immune score, stromal score, and enhanced tumor purity score than low-risk cohort (Fig. 5C). In our analysis of immunocytic infiltration, we focused on CD8+ T cells, which were observed infiltrating at higher levels in the high-risk cohort versus the low-risk cohort. Additionally, we observed the same infiltration pattern in macrophages M0 and M1 (Fig. 5D). The high-risk cohort's MHC molecule expression levels presented a notable reduction, as seen in Fig. 5E. Finally, we detected the expression patterns of common immunotherapeutic targets in LUAD treatment and discovered notable discrepancy between the high and low-risk cohorts (Fig. 5F).

### Association of angiogenic activity, mesenchymal EMT, tumorigenic cytokines, stemness scores and TSIs

**Figure 6:**
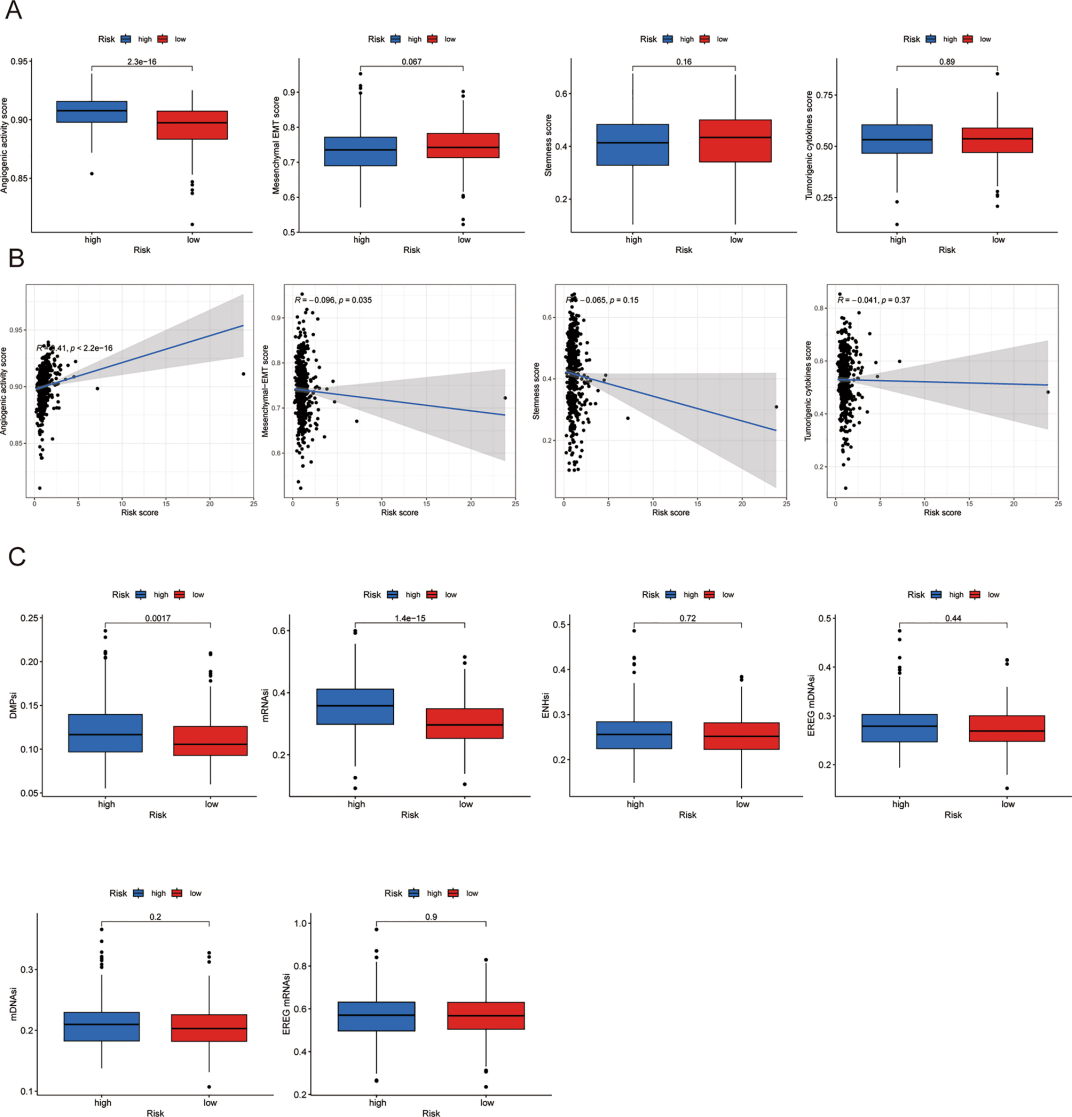
(**A**) Differences of angiogenic activity, mesenchymal-EMT, tumorigenic cytokines and stemness scores between the high- and low-risk groups. (**B**) The correlation of the risk score and angiogenic activity, mesenchymal-EMT, tumourigenic cytokines and stemness scores. (**C**) Differences of TSIs between the high and low risk groups.

In our former molecular sub-type analysis, we identified associations between different clusters and specific biological features, including angiogenic activity, mesenchymal EMT, tumorigenic cytokines, and stemness scores. For further study of this relationship, we calculated these scores for LUAD patients and compared them between the high-risk and low-risk cohort. Figure 6A indicated that the high-risk cohort presented increased angiogenic activity, however, no statistically significant differences were observed in the expression of mesenchymal EMT, tumourigenic cytokines and stemness scores. In Fig. 6B, we plotted the association of the risk score with the four indicators, revealing a positive relationship between the risk score and angiogenic activity score (R = 0.41, p < 9.43e−07), mesenchymal EMT score (R = − 0.096, p = 0.035), stemness score (R = − 0.065, p = 0.15), and tumorigenic cytokine score (R = − 0.041, p = 0.37). Furthermore, we also explored the association between the risk score and TSIs. Our investigation into the association between the risk score and TSIs revealed that the high-risk group exhibited higher levels of DMPsi, mRNAsi, EHNsi, EREG-mDNAsi, mDNAsi, and EREG-mRNAsi than the low-risk group. However, only DMPsi and mRNAsi presented notably significant differences (P < 0.05), as shown in Fig. 6C.

### Gene mutation analysis and TMB according to signature

**Figure 7:**
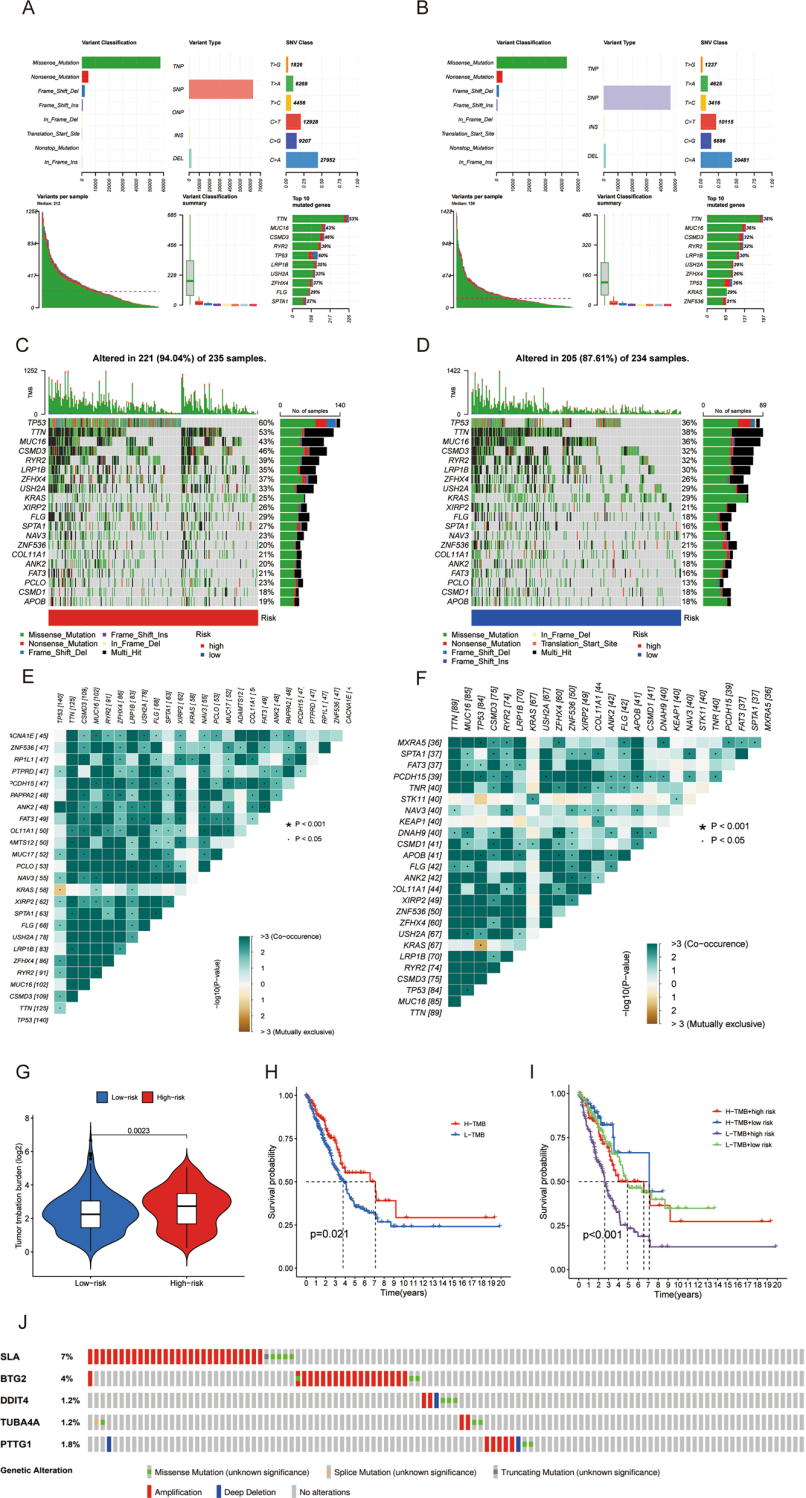
Characteristics of somatic mutations. The overall mutation profile of LUAD in the high-risk group (**A**) and the low-risk group (**B**) Waterfall maps of the somatic mutations in the high-risk group (**C**) and the low-risk group (**D**). Heatmap of co-occurrence and mutually exclusive mutations of the differently mutated genes in the high-risk group (**E**) and the low-risk group (**F**). (**G**) Differential expression levels of TMB between low-risk and high-risk groups. (**H**) The Kaplan–Meier curves for the low-TMB and high-TMB groups. (**I**) The Kaplan–Meier analysis curves for the patients stratified by risk scores and TMB. (**J**) Mutation rates of five genes (SLA, BTG2, DDIT4, TUBA4A, and PTTG1) in LUAD patients based on cBioPortal database.

Figure 7A,B displays the overall mutation profiles of LUAD in high- and low-risk cohorts. TP53, TTN, MUC16, CSMD3, and RYR2 emerged as the most frequently mutated genes in risk groups, presenting a higher mutation in the high-risk cohort (Fig. 7C,D). According to the high-risk cohort, we observed gene mutation co-occurrence among most genes, indicating that multiple correlated gene mutations may coexist, providing insight into tumor sample genetic changes and cancer development mechanisms. Additionally, mutually exclusive KRAS-TP53 mutations were identified in the high-risk cohort, suggesting that these gene mutations are unlikely to co-occur in the same sample (Fig. 7E). Figure 7F revealed that he low-risk cohort also exhibited mutual exclusivity and co-occurrence of gene mutations. These findings offered valuable information to explore gene mutation mechanisms in LUAD targeted therapies. Accoring to Fig. 7G, we compared tumor mutation burden (TMB) levels between the two cohorts and found a significantly higher TMB in the high-risk cohort compared to the low-risk cohort. Kaplan–Meier curves revealed that the high-TMB cohort had a better prognosis than the low-TMB cohort (P = 0.021) (Fig. 7H). After integrating the former risk signature, the low-risk + high-TMB cohort demonstrated a notably promising prognosis than other three cohorts (P < 0.001) (Fig. 7I). Lastly, we assessed the mutation patterns of the five signature genes and discovered notably higher mutation rates in SLA and BTG2 than in DDIT4, TUBA4A and PTTG1 (as illustrated in Fig. 7J).

### Prediction of the chemotherapy sensitivity analysis

**Figure 8:**
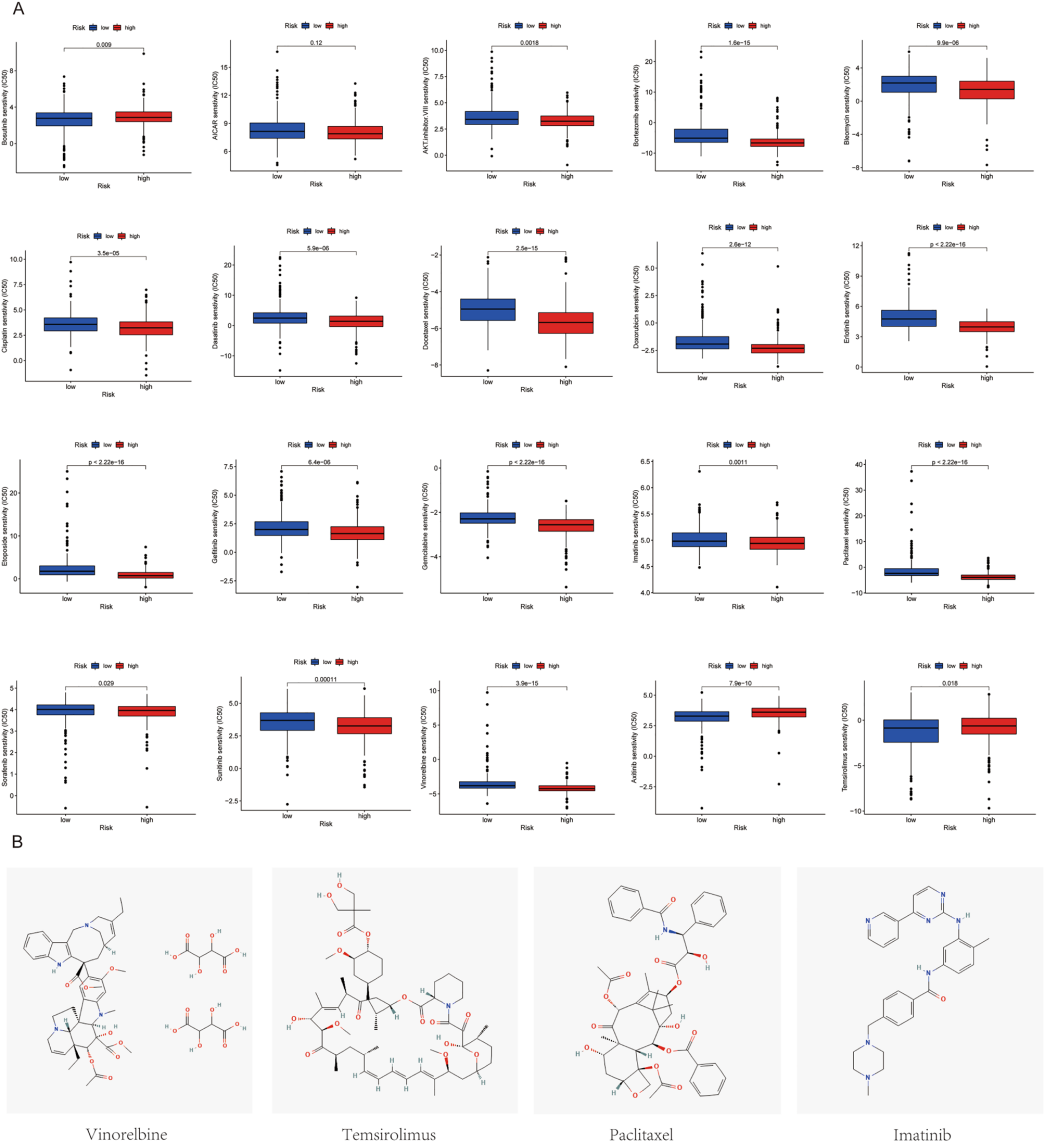
Evaluation of drug sensitivity. (**A**) The comparisons in chemotherapy response of common chemotherapy drugs between the high- and low-risk groups. (**B**) The 2D structure of most four common drugs used in LUAD chemotherapy.

We further employed "pRRophetic" to investigate differences in IC50 expression of chemotherapy medicines between low-risk and high-risk cohorts (Fig. 8A). Our findings revealed that LUAD in the high-risk cohort presented decreased IC50 values of anticancer drugs such as AICAR, AKT.inhibitor.VIII, bleomycin, bortezomib, bosutinib, cisplatin, dasatinib, docetaxel, doxorubicin, erlotinib, etoposide, gefitinib, gemcitabine, imatinib, paclitaxel, sorafenib, sunitinib, and vinorelbine. Low-risk patients showed lower IC50 values for anticancer medications such axitinib and temsirolimus. We showcased the 2D structures of the four most prevalent chemotherapy drugs—vinorelbine, temsirolimus, paclitaxel, and imatinib—using the PubChem database (Fig. 8B). Our results demonstrated that the risk model identified in this study may have clinical utility as a predictor of anticancer drug selection in patients with LUAD.

### Verification of the mRNA and protein expression in LUAD

**Figure 9:**
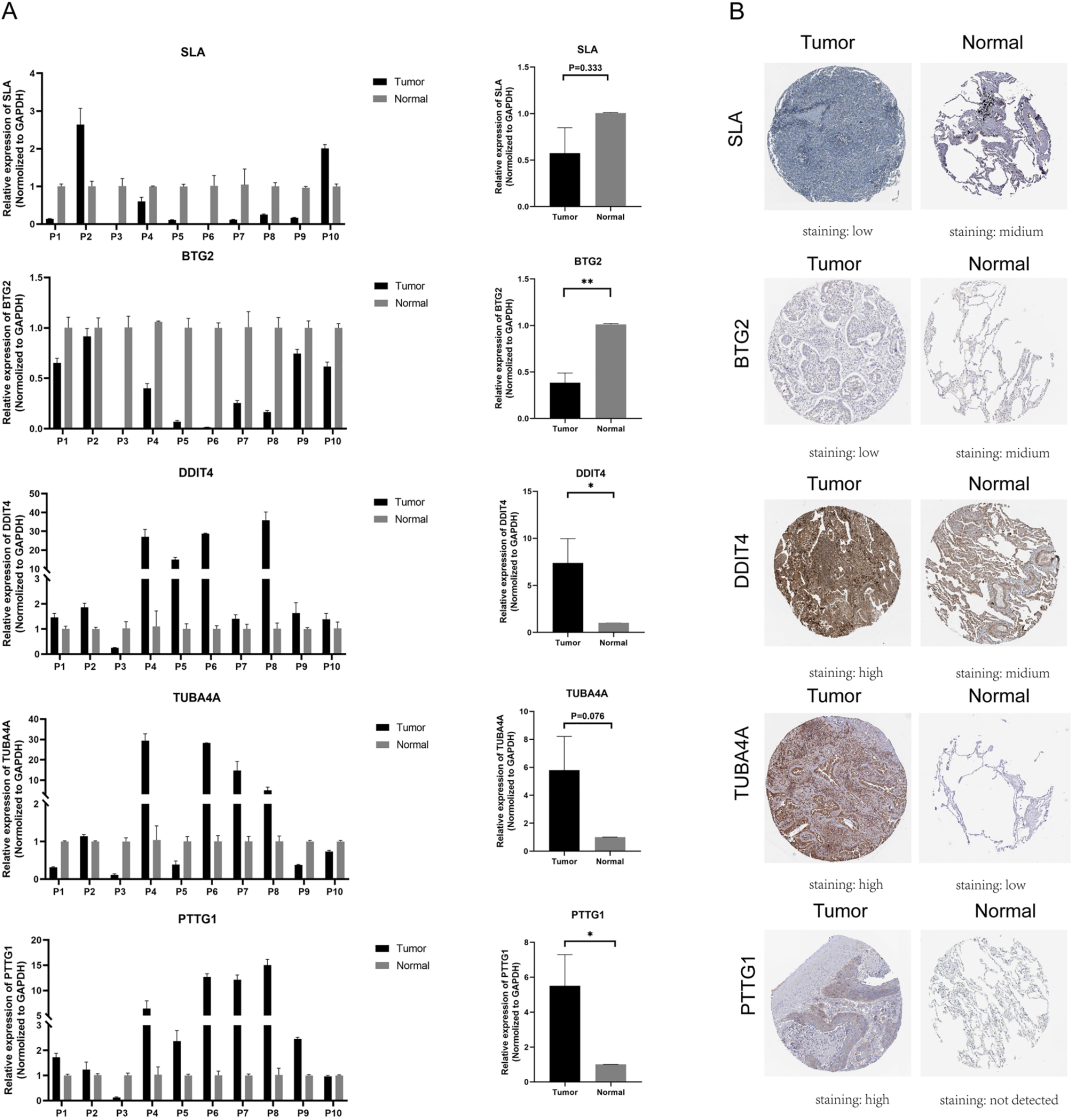
(**A**) Evaluation of the expression levels of SLA, BTG2, DDIT4, TUBA4A, and PTTG1 between normal lung specimens (n = 10) and LUAD specimens (n = 10) employing PCR analysis. All data are depicted as means ± SD. (**B**) Protein expression profiles of SLA, BTG2, DDIT4, TUBA4A, and PTTG1 in LUAD tissues compared to normal tissues, as obtained from the HPA database.

The RT-qPCR assay was employed to further elucidate the expression patterns of the five candidate genes in normal and LUAD tissues. We collected 10 pairs of LUAD tumor samples and their corresponding adjacent normal tissues. As depicted in Fig. 9A, the expression levels of DDIT4, TUBA4A, and PTTG1 were elevated in LUAD tissues compared to normal lung tissues, while the expression levels of SLA and BTG2 were diminished in LUAD tissues. This result was consistent with the expression of these five genes in TCGA (Supplementary Fig. 2). Additionally, the protein expression levels of SLA, BTG2, DDIT4, TUBA4A, and PTTG1 in LUAD tumor tissues and normal tissues were investigated using the Human Protein Atlas (HPA) database. The protein levels of DDIT4, TUBA4A, and PTTG1 were markedly elevated in tumor tissues, whereas SLA and BTG2 were notably diminished compared to normal tissues (Fig. 9B).

## Discussion

Immunotherapy has achieved notable success in treating patients with advanced tumors and is emerging as a potent clinical strategy for cancer treatment^30–32^. However, the clinical application of immunotherapy still faces significant challenges, including variability in effectiveness, drug resistance, side effects, and a lack of biomarkers^33–36^. The absence of dependable predictive markers is a significant contributing factor to these limitations. Single cell sequencing technology serves as a potent approach for examining cancer heterogeneity and distinct cellular subpopulations, which can be crucial for recognizing potential therapy targets^37^. The current study aimed to analyze combined single cell data and bulk data from TCGA to uncover cellular communication patterns between immune and tumor cells in LUAD patients. We identified marker genes of T cell in LUAD and developed a 5-gene prognostic signature, which was validated using the GEO cohort. GO and KEGG enrichment analyses indicated that the marker genes are primarily enriched in immune-related pathways. Furthermore, notable discrepancies were observed in immune scores, stromal scores, Immunocytes infiltration, immune checkpoints expression patterns, and somatic mutations situations between risk sub-cohorts. These findings offer new insights for precision treatment and personalization strategies for LUAD patients.

In our study, the prognostic signature comprised five T cell marker genes: SLA, DDIT4, TUBA4A, PTTG1, and BTG2. Previous research has shown that overexpression of SLA inhibits intrahepatic cholangiocarcinoma (IHCC) cell growth and induces cell cycle arrest, suggesting a tumor-suppressive role for SLA in IHCC progression^38^. Additionally, SRC-like adapter protein 2 (SLAP2) has been identified as a negative regulator of KIT-D816V-mediated oncogenic transformation^39^. Elevated DDIT4 expression has been linked to reduced overall survival in LUAD patients. Moreover, the expression of the DDIT4 gene is notably enhanced in hypoxic conditions than in normoxic ones, indicating that DDIT4 may have a significant role in the hypoxic microenvironment of tumor tissues^40^. As TUBA4A is a component of microtubules, the effectiveness of microtubule-targeting drugs (e.g., paclitaxel-like drugs) may be impacted in lung cancer treatment. Studies have shown that in lung cancer cells, mutations in TUBA4A can result in resistance to microtubule-targeting drugs^41^. Many tumor types exhibited increased PTTG1 expression, which is involved in controlling the development and spread of malignancies. In vitro investigations with lung cancer cell lines revealed that silencing PTTG1 inhibited cell proliferation and invasion. Additionally, PTTG1 knockdown impaired the invasive ability of in situ LLC tumor-bearing mice, promoting a shift in the balance of IR-induced immune response towards active immunity^42,43^. Reducing the expression of NUSAP1 leads to an increase in B-cell translocation gene 2 (BTG2) expression, which in turn promoted apoptosis and inhibited cell growth, migration, and invasion in NSCLC cells^44^. In the prognostic model we developed, SLA and BTG2 were identified as protective factors, while DDIT4, TUBA4A, and PTTG1 were considered risk factors. Significantly, we further verified the mRNA expression levels of SLA, BTG2, DDIT4, TUBA4A, and PTTG1 by examining clinical specimens. Concurrently, we also confirmed the protein expression of these genes through the HPA database. These results provided further evidence to support the bioinformatics analysis findings, and this multi-faceted approach simultaneously strengthens the overall conclusions while demonstrating the potential clinical relevance of the results.

Moreover, given the pivotal position of tumor-infiltrating immune cells in TME for tumor development and their significant impact on prognosis, we next compared the levels of immune cell infiltration between risk cohorts using ESTIMATE and CIBERSORT algorithms^45,46^. Overall, tumors in the high-risk cohort presented decreased levels of infiltrating immunological cells and reduced Immunological function, indicating that these high-risk group tumors are characterized as "cold tumors" with low anti-tumor activity^47,48^. The decreased immune cell infiltration may enable tumor cells to evade Immunosurveillance and promote cancer development. This phenomenon may account for the notably lower survival rate of high-risk LUAD patients. Our findings revealed that immune checkpoint-related genes (CTLA4, ROS1, ALK, BRAF, RET) commonly found in the increased expression of LUAD in the low-risk sample indicated that immunotherapy may be more appropriate for this cohort. Lastly, HLA, the expression product of the major human histocompatibility complex, is an antigen-presenting molecule that regulates the Immunological response in pulmonary adenocarcinoma^49–51^. Our study observed that the majority of HLA family genes were extensively expressed in low-risk cohort, suggesting a more active local Immunological response. Taken together, patients of the low-risk cohort displayed increased immune cellular infiltration and Immunological response, indicating that they may be more responsive to immunotherapy.

We then explored the association between TMB and risk models. The frequencies of alterations in TP53, TTN, MUC16, CSMD3, and RYR2 were notably distinct between the high and low-risk cohorts. TP53 functions as a tumor suppressor, responsible for controlling cell growth, DNA repair, and apoptosis. Mutations in the TP53 gene can lead to the loss of these functions, resulting in uncontrolled cell growth, genomic instability, and resistance to apoptosis, which ultimately leads to the development and progression of tumor, including LUAD^52,53^. MUC16 mutations may be associated with a higher tumor mutation load (TML), better survival outcomes, immune response, and cell cycle pathways^54^. These results could be potentially applicable for optimizing immunotherapy in tumor patients. CSMD3 mutations were found to be highly associated with increased TMB and poor clinical prognosis and may be used as indicators to predict prognosis and choose immunotherapy regimens^55^. Patients with RYR2 mutations in esophageal cancer exhibit higher tumor mutation load (TMB), better prognosis, and enhanced immune checkpoint expression^56^. These studies suggest that investigating mutational characteristics may lead to improved selection of immunotherapy for individual patients.

To better optimize chemotherapy treatment regimens for LUAD, we conducted drug sensitivity analyses on subgroups. We examined the 20 most common anticancer drugs targeting LUAD among the low-risk and high-risk cohorts. Our findings revealed that the high-risk cohort was sensitive to 18 anticancer drugs, including AICAR, AKT.inhibitor.VIII, bleomycin, bortezomib, bosutinib, cisplatin, dasatinib, docetaxel, doxorubicin, erlotinib, etoposide, gefitinib, gemcitabine, imatinib, paclitaxel, sorafenib, sunitinib, and vinorelbine. The low-risk group, however, was susceptible to two anticancer medications, notably axitinib and temsirolimus, which served as a clinical benchmark for the choice of chemotherapy agents. We intend to further explore the clinical importance of these medications in LUAD patients in our follow-up study.

While this study uses T-cell marker genes as a starting point to advance the development of novel therapeutic approaches for LUAD, it does have some limitations. Firstly, the number of scRNA-seq samples available in public databases is limited, which may affect the study's persuasiveness. To validate our findings, further in-depth in vivo experiments are required. Secondly, drug sensitivity should be further confirmed through cellular experiments to ensure accuracy. In future research, it is essential to explore the potential mechanisms linking T-cell immunity and LUAD prognosis to improve our understanding of the disease and develop more effective therapy strategies.

## Methods

### Data acquisition

Dataset GSE148071's single-cell RNA sequencing profile, which includes 60,288 individual cells from 42 patients, was sourced from the Gene Expression Omnibus (GEO, https://www.ncbi.nlm.nih.gov/geo/). Additionally, RNA-seq expression information and clinical data for LUAD patients (https://portal.gdc.cancer.gov/projects/TCGA-LUAD) were extracted from The Cancer Genome Atlas (TCGA, https://cancergenome.nih.gov/), amassing a total of 504 LUAD specimens and 54 healthy lung tissues from the database. To authenticate the predictive capacity of the model, transcriptomic and clinical records of dataset GSE13213, comprising 117 LUAD samples, were obtained from the GEO.

### Single-cell and bulk RNA-seq data manipulation

By leveraging the "Seurat" package in R (version 4.2.0), a total of 60,288 cells were meticulously classified into suitable clusters, utilizing the resolution parameter of 0.4. The insights were subsequently deciphered through the t-distributed stochastic neighbor embedding (t-SNE) method for dimensionality reduction. Additionally, the "Cellchat" package (version 1.5.0) was employed to explore cellular crosstalk. With respect to the TCGA RNA-seq data, differentially expressed genes (DEGs) were ascertained by juxtaposing 54 normal and 504 LUAD tissue samples using the DESeq2 R package, and the filter criteria was FDR < 0.05 and |log2FC| > 1 as threshold criteria. Functional enrichment analysis for the intersection of DEGs and T cell-marker genes, including gene ontology (GO) analysis and KEGG analysis, was performed using the clusterProfiler R package. Threshold values were set as an adjusted p < 0.05.

### Identifying T cell marker genes using scRNA-seq analysis

To ensure high-quality single cell sequencing objects, two filtering criteria were applied to each cell within the raw Seurat: cells displaying gene expression levels ranging from 200 to 7000 were included, while cells with over 20% mitochondrial genes were excluded. The "Seurat" package was initially employed to normalize data. The "Harmony" tool was used to mitigate batch effects among samples. Subsequently, the "RunPCA" function within the "Seurat" R package was utilized to conduct principal component analysis (PCA) for dimensionality reduction of the scRNA-seq data. The "FindNeighbors" and "FindClusters" were harnessed for cell aggregation assessment. The k-nearest neighbor graph was developed by the "FindNeighbors" to determine each cell's closest neighbors. TSNE was then executed via the "RunTSNE" function. Cell markers from the CellMarker website (http://biocc.hrbmu.edu.cn/CellMarker/) were required for cell type identification and scHCL (an R package for large-scale data derived from the scHCL online function Human Cell Landscape). CellChat, a database encompassing receptor-ligand interactions, aided in the analysis of cell-to-cell signaling pathways. To extract critical cell–cell interactions between immune and cancer cells, receptor-ligand pairs were chosen for comprehensive examination.

### Molecular subtype analysis

Intersection of T cell marker genes, derived from single cell data, and DEGs, originating from TCGA differential expression analysis, was performed. Subsequently, the R package "ConsensusClusterPlus" facilitated cluster analysis, identifying the molecular subtype of lung adenocarcinoma. To assess the prognostic variations among the sub-clusters, Kaplan–Meier (K–M) analysis was used. Relationships between subtypes and clinical information are depicted in heatmaps and appraised with chi-square tests.

### Generation and verification of prognostic signature according to T cell marker genes

Utilizing multivariate Cox regression analysis via “coxph” function of “survival” package, independent genes for LUAD were discerned, leading to the development of a prognostic model. The coefficients of the chosen genes were displayed through Excel software. A risk model was formulated by combining gene mRNA expression linearly with pertinent risk coefficients. The equation was used to calculate the risk score for each patient. Risk score = coefficient (mRNA) * expression (mRNA). Using the median risk score as the cutoff, the training cohort was divided into low- and high-risk groups. To substantiate the prediction efficacy of our signature, the AUC value was determined via "survivalROC" package, while the K-M analysis underpinned survival analysis. GSE13213 was employed to validate the prognostic model. Cox analyses were harnessed to ascertain the signature's role as an independent risk element. Drawing from clinicopathological information, a correlational analyses between correlational analyses between clinical attributes and risk scores was undertaken, succeeded by stratified analysis and nomogram build-up. Calibration diagrams facilitated comparisons between the congruence of predictive proximity for 1-, 3-, and 5-year mortality rates and observed outcomes.

### Immune landscape exploration

Three Immune-associated arithmetics were implemented to investigate immune status across molecular subtypes as well as between high- and low-risk groups. Single-sample gene set enrichment analysis (ssGSEA) was further performed to explore the activity of immunocytes and Immunological functions within each sample. The ESTIMATE algorithm facilitated the calculation of immune scores, stromal scores, estimate scores, and tumor purity based on the proportions of immune and stromal cells. Using the CIBERSORT algorithm, each LUAD sample's Immunocytes population's makeup was identified. Additionally, the expression level of MHC molecules was compared according to aggregation analysis and signatures. With respect to immune checkpoints, common immunoinhibitory molecules were initially contrasted following clusters and risk levels.

### Tumor-related scores and tumor stemness indices (TSIs) exploration

According to earlier studies, tumor patients with poor prognoses have higher tumor-related scores, such as those for angiogenic activity, mesenchymal-epithelial-mesenchymal transition (EMT), tumourigenic cytokine, and stemness. We calculated tumor-related scores in each tumor sample using the ssGSEA algorithm. Due to the correlation between dynamic biological processes and altered tumour devitrification in stem cells, TSIs were found in both the high risk and low risk cohorts.

### Gene mutation landscape

Utilizing gene somatic mutation profiles on TCGA, we next performed the mutation analysis via the "maftools" R package. TMB was calculated for individual patients and compared between high- and low-risk cohorts. Additionally, we performed survival analysis to examine the association between TMB scores and patient outcomes, and somatic mutations in selected candidate genes were illustrated according to cBioPortal website.

### Chemotherapy sensitivity prediction

In evaluating the predictive signature's significance for forecasting chemotherapy sensitivity in LUAD, the "pRRophetic" R package was employed to ascertain the half-maximal inhibitory concentration (IC50) of principal chemotherapeutic candidates utilized in LUAD treatment. The two-dimensional structural illustrations of these prospective pharmaceuticals were procured from the PubChem database.

### Collection of LUAD patients and tissue specimens

In this investigation, we procured 10 matched pairs of LUAD tumor and corresponding adjacent normal lung tissues from the Affiliated People's Hospital of Shanxi Medical University between January 2021 and May 2022. These samples were promptly frozen and preserved at − 80 °C for subsequent utilization in real-time quantitative polymerase chain reaction (RT-qPCR) experiments. To guarantee adherence to ethical standards, the Research Ethics Committee of the Affiliated People’s Hospital of Shanxi Medical University sanctioned this study (No. 2022-111), which complied with the principles outlined in the Declaration of Helsinki. Moreover, all participants furnished written informed consent prior to their involvement in this research.

### RT-qPCR validation

Total RNA was isolated using TRIzol reagent (Takara, Japan). Subsequently, first-strand cDNA was synthesized from 1 μg of total RNA employing the PrimeScript RT Reagent Kit with gDNA Eraser (Takara, Japan). SYBR Green (Takara, Japan) served as the molecular probe. qRT-PCR analysis of specific cDNAs was conducted utilizing the ABI PRISM 7500 detection system (Applied Biosystems). The cycling parameters entailed 30 s of polymerase activation at 95 °C, followed by 40 cycles at 95 °C for 5 s and 60 °C for 34 s. GAPDH functioned as the internal loading control, and the relative expression levels were computed using the 2^−ΔΔCT^ method for relative quantification. All primer sequences for GAPDH, SLA, BTG2, DDIT4, TUBA4A, and PTTG1 are provided in Supplementary Table 2.

### Protein expression validation using the Human Protein Atlas database (HPA)

The Human Protein Atlas (HPA) database provides insights into protein expression profiles across diseased and healthy tissues. In our investigation, we corroborated the expression of candidate genes (SLA, BTG2, DDIT4, TUBA4A, and PTTG1) between LUAD and normal lung tissues through the examination of immunohistochemical data available in the HPA database.

### Statistical analysis

All the statistical analyses were conducted using R software. Wilcoxon test was performed to analyze differences for PCR experiments. We set the statistical significance threshold to a P value less than 0.05 in this study. To minimize bias in the study, two independent researchers performed literature searches, data extraction, and analysis.

### Supplementary Information

Supplementary Legends.

Supplementary Figure 1.

Supplementary Figure 2.

Supplementary Table 1.

Supplementary Table 2.

## Data Availability

Our results utilized publicly available data from the TCGA and the GEO. The accession numbers for the utilized GEO datasets are GSE148071 and GSE13213.
