# Supplementary material for: Integrative analysis of single-cell and bulk RNA-sequencing data revealed T cell marker genes based molecular sub-types and a prognostic signature in lung adenocarcinoma
Source: Sci Rep. 2024 Jan 10;14:964. doi: 10.1038/s41598-023-50787-w (PMC10781781; doi:10.1038/s41598-023-50787-w)
Supplement: Supplementary file 1 — Supplementary Legends. [file 41598_2023_50787_MOESM1_ESM.docx]

Supplementary Figure legends

Supplementary Figure 1. The workflow of the current study.

Supplementary Figure 2. The expression of BTG2, PTTG1, TUBA4A, DDIT4, and SLA in TCGA.
