# Supplementary material for: Integrative analysis of single-cell and bulk RNA-sequencing data revealed T cell marker genes based molecular sub-types and a prognostic signature in lung adenocarcinoma
Source: Sci Rep. 2024 Jan 10;14:964. doi: 10.1038/s41598-023-50787-w (PMC10781781; doi:10.1038/s41598-023-50787-w)
Supplement: Supplementary file 2 — Supplementary Figure 1. [file 41598_2023_50787_MOESM2_ESM.pdf]

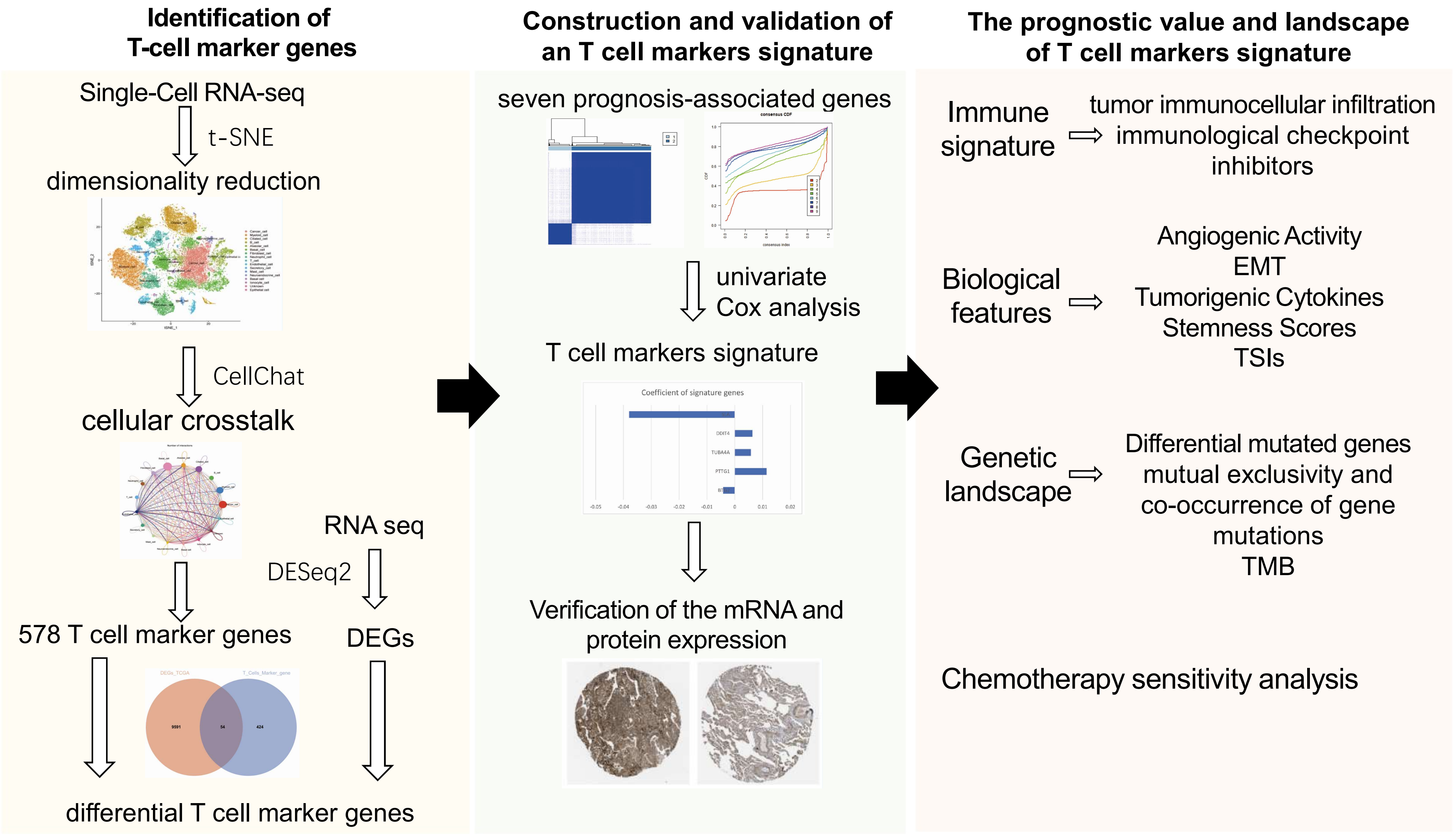
Identification of T-cell marker genes
Single-Cell RNA-seq
t-SNE
dimensionality reduction
CellChat
cellular crosstalk
RNA seq
DESeq2
578 T cell marker genes
DEGs
9591
54
424
differential T cell marker genes
Construction and validation of an T cell markers signature
seven prognosis-associated genes
univariate Cox analysis
T cell markers signature
Coefficient of signature genes
DDIT4
TUBA4A
PTTG1
Verification of the mRNA and protein expression
The prognostic value and landscape of T cell markers signature
Immune signature
tumor immunocellular infiltration
immunological checkpoint inhibitors
Biological features
Angiogenic Activity
EMT
Tumorigenic Cytokines
Stemness Scores
TSIs
Genetic landscape
Differential mutated genes
mutual exclusivity and co-occurrence of gene mutations
TMB
Chemotherapy sensitivity analysis
