# Supplementary figures and images for: Integrative analysis of single-cell and bulk RNA-sequencing data revealed T cell marker genes based molecular sub-types and a prognostic signature in lung adenocarcinoma
Source: Sci Rep. 2024 Jan 10;14:964. doi: 10.1038/s41598-023-50787-w (PMC10781781; doi:10.1038/s41598-023-50787-w)

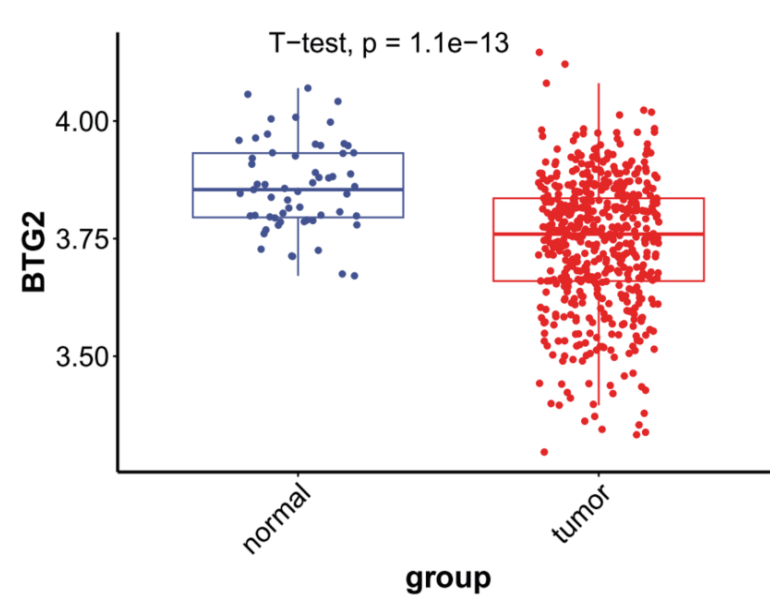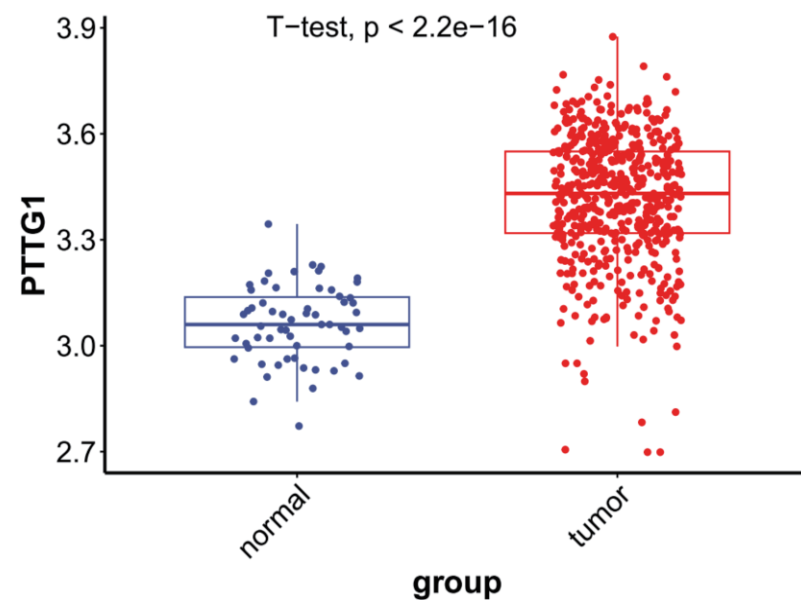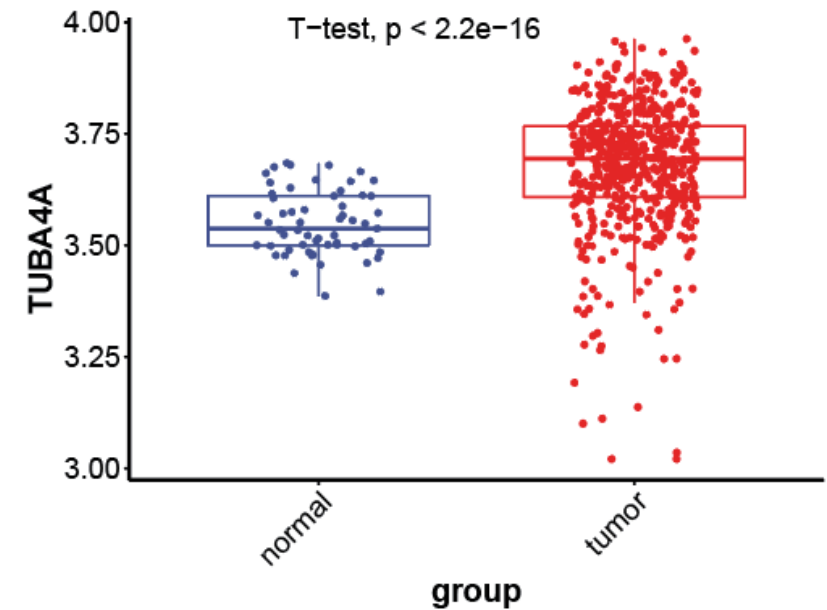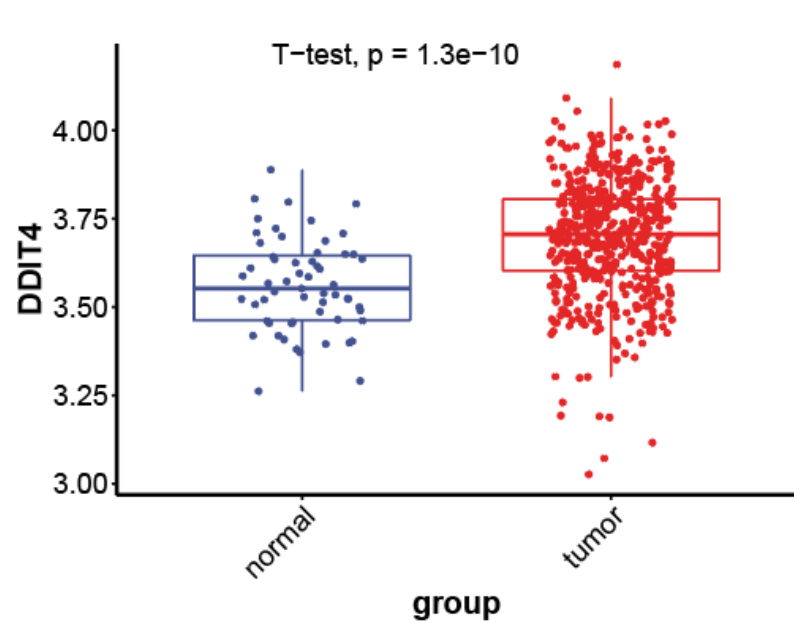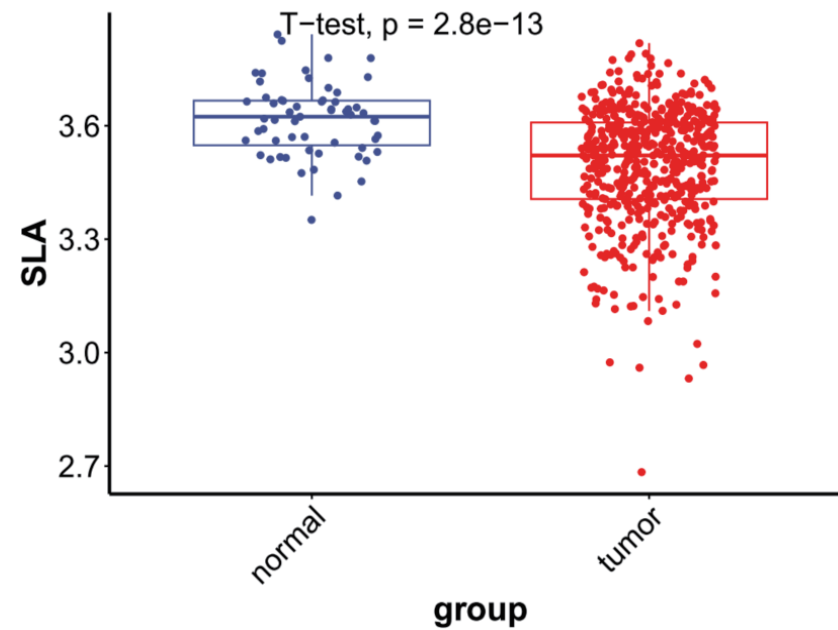

Supplement: Supplementary file 3 — Supplementary Figure 2. [file 41598_2023_50787_MOESM3_ESM.pdf]
